# Molecular and cellular factors determining the functional pleiotropy of cytokines

**DOI:** 10.1111/febs.16420

**Published:** 2022-03-14

**Authors:** Alison McFarlane, Elizabeth Pohler, Ignacio Moraga

**Affiliations:** ^1^ Division of Cell Signalling and Immunology School of Life Sciences University of Dundee UK

**Keywords:** cytokine signalling, endosomes signalling, JAK/STAT signalling pathway, pleiotropy, protein engineering

## Abstract

Cytokines are soluble factors vital for mammalian physiology. Cytokines elicit highly pleiotropic activities, characterized by their ability to induce a wide spectrum of functional responses in a diverse range of cell subsets, which makes their study very challenging. Cytokines activate signalling via receptor dimerization/oligomerization, triggering activation of the JAK (Janus kinase)/STAT (signal transducer and activator of transcription) signalling pathway. Given the strong crosstalk and shared usage of key components of cytokine signalling pathways, a long‐standing question in the field pertains to how functional diversity is achieved by cytokines. Here, we discuss how biophysical – for example, ligand‐receptor binding affinity and topology – and cellular – for example, receptor, JAK and STAT protein levels, endosomal compartment – parameters contribute to the modulation and diversification of cytokine responses. We review how these parameters ultimately converge into a common mechanism to fine‐tune cytokine signalling that involves the control of the number of Tyr residues phosphorylated in the receptor intracellular domain upon cytokine stimulation. This results in different kinetics of STAT activation, and induction of specific gene expression programs, ensuring the generation of functional diversity by cytokines using a limited set of signalling intermediaries. We describe how these first principles of cytokine signalling have been exploited using protein engineering to design cytokine variants with more specific and less toxic responses for immunotherapy.

## Graphical abstract

Cytokines produce a wide range of biological responses despite using a very minimal set of effector signalling molecules. Here, we review how molecular and cellular parameters ultimately converge into a common mechanism of cytokine signalling and how these can be exploited for more targeted therapies.

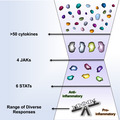

**Abbreviations**
CDK: cyclin‐dependant kinase
CMV: cytomegalovirus
DARPins: designed ankyrin repeat proteins
DN: dominant negative
EBV: Epstein–Barr
ECD: extracellular domain
EGFR: epidermal growth factor receptor
Epo: erythropoietin
EpoR: erythropoietin receptor
ER: endoplasmic reticulum
ESCRT: endosomal sorting complex required for transport complex
G‐CSF: granulocyte colony‐stimulating factor
G‐CSFR: granulocyte colony‐stimulating factor receptor
GH: growth hormone
GHR: growth hormone receptor
GM‐CSF: granulocyte–macrophage colony‐stimulating factor
GP130: glycoprotein‐130
GPCR: G‐protein‐coupled receptors
GPL: glycoprotein‐130 like
HIV: human immunodeficiency virus
Hrs: hepatocyte growth factor‐regulated tyrosine kinase substrate
ICD: intracellular domain
IFN: interferon
IFNAR: type 1 interferon receptor
IL: interleukin
JAK: Janus kinase
LIFR: leukaemia inhibitory factor receptor
MHC: major histocompatibility complex
mTOR: mammalian target of rapamycin
NK: natural killer
OSMR: Oncostatin‐M receptor (in the text you call this OSM‐R)
Ph– MPNs: Philadelphia chromosome–negative myeloproliferative neoplasms
PI(3)P: phosphatidylinositol 3‐phosphate
RTK: receptor tyrosine kinases
Ser: serine
STAM: signal‐transducing adaptor molecule
STAT: signal transducer and activator of transcription
T reg: T regulatory
TCR: T cell receptor
TGN: Trans‐Golgi network
TM: transmembrane
Tpo: thrombopoietin
TYK2: tyrosine kinase 2
Tyr: tyrosine
vIL: viral interleukin
γc: gamma chain

## Introduction

Four‐helical cytokines are a large family of secreted proteins vital for mid‐ and long‐range cell‐to‐cell communication, which regulate all aspects of mammalian physiology [1, 2, 3]. Cytokines are implicated in the pathologies of many human diseases, including inflammatory disorders and cancer, highlighting their importance in human health [4, 5]. Mutations in IL‐2Rγ, a receptor subunit used by IL‐2, IL‐7, IL‐15 and other key immuno‐cytokines, leads to severe combined immunodeficiency [6, 7]. High levels of IL‐6 are found in several chronic inflammatory syndromes and correlate with disease severity [8]. Defects in IL‐10 signalling leads to early‐onset colitis and Crohn’s disease [9]. Despite the great therapeutic potential of this family, very few cytokines have been successfully translated to the clinic [e.g. IL‐2 (Aldesleukin), IFNα2 (Roferon‐A), IL‐11 (Neumega)], due to high systemic toxicity and off‐target effects.

Cytokines trigger signalling by ligand‐induced assembly of cell surface receptors [10, 11, 12], resulting in the activation of receptor‐associated Tyr kinases of the Janus Kinase (JAK) family [13, 14]. JAKs, in turn, phosphorylate Tyr residues in the intracellular domain of cytokine‐receptors which the signal transducer and activator of transcription (STAT) transcription factors bind to [15, 16]. Once bound, STATs are phosphorylated by JAKs on Tyr, form homo‐ or hetero‐dimers and translocate to the nucleus where they induce the expression of specific gene programs, which ultimately determine cell fate [17, 18]. In addition to STATs, cytokines can also engage non‐STAT signalling, which help them to fine‐tune and diversify their responses. For example, IL‐2 and interferons (IFNs) can activate the mammalian target of rapamycin (mTOR) pathway, which contributes to the translation of the gene programs elicited by these cytokines [7, 19].

Cytokines exhibit two features that have made their study extremely challenging: (a) Functional pleiotropy – or the ability of one cytokine to elicit a wide spectrum of functional responses in a diverse range of cell subsets [3, 20]. For instance, IL‐10 supresses the production of pro‐inflammatory cytokines from monocytes and macrophages, but potentiates the effector functions of CD8+ T cells [21, 22]. (b) Functional redundancy – defined by the overlapping activities exhibited by groups of cytokines. For example, IL‐4 and IL‐13 share a common surface receptor to signal and elicit highly overlapping responses [23, 24]. These two cytokine properties stem from the loose nature of the cytokine‐receptor complex identity and from the relatively low number of JAKs (JAK1‐3 and TYK2) and STATs (STAT1‐6) used by cytokines. On the one hand, a given cytokine can engage multiple receptor complexes, leading to activation of different JAK/STAT pathways and induction of differential responses [25, 26, 27]. On the other hand, some cytokine‐receptors, such as GP130, IL‐10Rβ, γc and common beta chain are engaged by multiple cytokines, acting as signalling hubs with overlapping functional properties [28, 29, 30, 31]. How cytokines achieve functional specificity, despite sharing receptors and signalling components, remains a long‐standing question in the cytokine field [32].

In the last 10 years, we have seen an explosion of new studies in different cytokine systems that have started to shed light onto the molecular principles underlying cytokine functional diversity [33, 34, 35]. One aspect that appears clear is that the identity of the signalling response activated by any given cytokine, at least when focused on JAK/STAT signalling, does not predict its biological properties. For instance, although IL‐6 and IL‐10 both activate STAT3, they elicit opposite immune responses, with IL‐10 promoting an anti‐inflammatory response and IL‐6 driving inflammation [17, 36]. Thus, additional parameters beyond JAK/STAT signalling identity must be engaged by cytokines and cells to ultimately define the nature of the response generated [37]. In this review, we will summarize recent advances in the field that paint a complex picture where multiple layers of regulation are used by cells to control and manipulate cytokine responses. This functional plasticity allows cells to adapt their responses to the environment where they reside. We will also discuss recent advances in biomolecular engineering techniques that have allowed the manipulation and decoupling of cytokine responses, providing us with a better understanding of cytokine complex biology and improving cytokine therapeutic potential.

## Cytokine‐receptor binding parameters contributing to signalling diversification

**Fig. 1:**
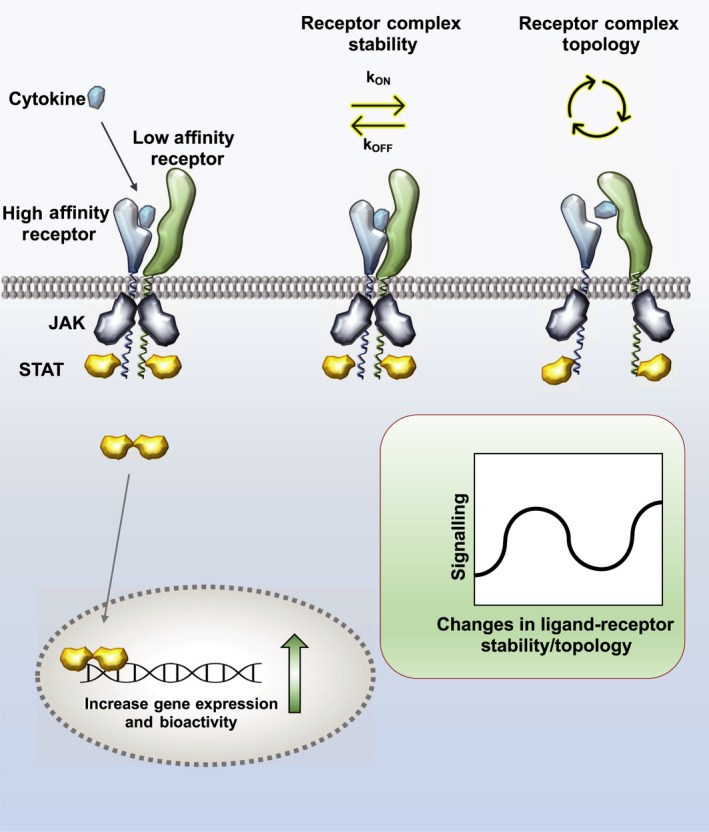
Biophysical determinants contributing to cytokine functional pleiotropy. In general terms, cytokines first bind a receptor subunit with high affinity, then in a second step, recruit a second subunit with low affinity to form the signalling complex. Upon dimerization, the receptor‐associated JAKs trans‐phosphorylate and activate each other, leading to the phosphorylation of tyrosines (Tyr) in the receptor intracellular domain. These phosphotyrosines act as docking sites for STATs, which upon binding are phosphorylated in Tyr by JAKs, form homo‐ or hetero‐dimers and translocate to the nucleus, where they induce the expression of specific gene programs. Any alteration that affects either the kinetics of the cytokine‐receptor complex formation or its geometry ultimately impacts downstream signalling and cytokine biological activities. This has been exploited by protein engineering to design more specific and less toxic cytokine variants.

Cytokines trigger signalling by dimerizing or oligomerizing receptors in the surface of responsive cells [10, 11, 12]. Generally, cytokines engage their receptors following a two‐step binding mechanism, with the cytokine first binding one of the receptor subunits with high affinity, and then in a second step, recruiting a second receptor subunit with lower affinity to the complex to initiate signalling [11, 38, 39]. This trimeric receptor complex represents the minimal entity required for signal activation, where at least two JAKs are juxtaposed to initiate the signalling cascade. However, cytokines can form complexes with larger complex stoichiometries, ranging from tetramers (e.g. IL‐2, IL‐15, IL‐12) to hexamers (e.g. IL‐10, IFNy, IL‐6, IL‐11) and dodecamers (e.g. GM‐CSF) [11, 31]. A leading hypothesis in the field to explain how cytokines achieve their large functional diversity is that receptor binding parameters, such as receptor binding geometry and affinity, can be interpreted by cells to initiate specific signalling programs that fine‐tune cytokine responses (Fig. 1) [33]. In agreement with this model, it is now well established that changes in cytokine‐receptor binding affinities impact cytokine receptor complex formation, kinetics and signalling [11, 38, 39]. However, how the different cytokine‐receptor complex half‐lives ultimately produce different signalling profiles that result in unique biological responses remains an open question. Similarly, the role of cytokine‐receptor binding geometry in functional diversification remains the subject of intense debate in the field. In this chapter, we will discuss advances in the field that have shed new light onto how cytokine‐receptor binding affinity and geometry may contribute to regulate cytokine responses.

## Cytokine‐receptor complex stability/kinetics and signalling

### Disconnect between cytokine‐receptor complex stability, signalling and bioactivity potency

Many studies have now reported a clear correlation between cytokine‐receptor complex stability and bioactivity potency as highlighted in Fig. 1 [3, 33, 39]. Long‐lived cytokine‐receptor complexes promote stronger responses than short‐lived complexes. However, the same pattern is not observed when cytokine signalling is analysed. In this instance, signalling remains largely unaffected at a wide range of complex stabilities, establishing a well‐known cytokine paradox – how comparable signalling programs engaged by multiple cytokines exert different biological potencies. In recent years, new evidence from different cytokine systems suggest that the number of STAT molecules activated over time, rather than the identity of the STAT protein engaged, ultimately determines the nature and potency of the cytokine response [40, 41].

This cytokine paradox was first identified in the type I interferon (IFN) system, where initial studies help to establish the theoretical and experimental framework to understand its molecular bases. The type I IFN family comprises more than 16 different interferon subtypes, which are produced in response to environmental stresses like viruses, playing a central role in mounting potent antiviral responses [29, 39, 42]. IFNs also have anti‐proliferative properties, making them highly relevant to fighting cancer [43]. Indeed, IFNs are currently approved to be used in the clinic to treat several forms of cancer and viral infections [44]. All type I IFN subtypes engage the same hetero‐dimeric surface receptor, comprised of IFNAR1 and IFNAR2 subunits, and activate very comparable signalling profiles [45, 46, 47, 48, 49]. Yet, when more complex activities are studied, for example, antiviral and anti‐proliferative activities, a broad range of potencies are found, highlighting this disconnect between early signalling events and late biological outcomes [47, 48, 49, 50]. Early alanine mutagenesis studies, focused on disrupting binding of IFNs to either IFNAR1 or IFNAR2 subunits, showed that overall decreases in binding affinity and complex stability resulted in a subsequent decrease in signal activation and biological activity potencies by IFNs [47, 51, 52, 53]. Interestingly, the same direct correlation was not observed when IFNs’ binding affinity and complex stabilities were increased. In this case, IFN signalling and antiviral potencies were not further increased by an enhancement in complex stability, suggesting that these two activities reach a plateau at binding affinities close to those exhibited by the wild‐type ligands [54, 55]. IFN anti‐proliferative activities, on the other hand, closely correlated with the stability of the IFN‐receptor complex, with IFN mutants forming more stable complexes triggering more potent responses than wild‐type IFNs [54, 55]. The relevance of IFN‐receptor complex stability for anti‐proliferative activity potency was further confirmed in studies where the overall stability of the receptor complex was maintained constant, but the relative affinities of IFN for either receptor subunits were either decreased or increased. Here again, the overall stability of the IFN‐receptor complex, rather than the individual affinities of IFN for either receptor subunits predicted IFN anti‐proliferative potencies [51, 56]. These studies for the first time highlighted that not all cytokines' bioactivities require the same threshold of signal activation to be induced, opening the door to engineering cytokine partial agonists with bias bioactivity profiles. Intriguingly, while type I IFNs' antiviral potency is saturated at endogenous receptor binding affinities, this is not the case for type III IFNs, where further enhancement in receptor binding affinity increases both antiviral and anti‐proliferative activities [57]. How long‐lived IFN‐receptor complexes elicit stronger anti‐proliferative responses than short‐lived IFN complexes, despite activating comparable signalling programs, remains an open question. Studies quantitatively analysing signalling responses by different IFN subtypes provide some rationale for this functional disconnection. These studies revealed a correlation between complex stability and signalling kinetics. Long‐lived receptor complexes triggered faster STAT activation kinetics than short‐lived complexes [56]. However, at the moment, we do not know how these observed changes in signalling kinetics contribute to fine‐tuning IFN responses. Further studies focused on understanding how STAT activation kinetics contribute to the induction of specific gene expression programs by different IFN subtypes will help to address this IFN functional paradigm.

These studies in the IFN system suggest that the half‐life of the cytokine‐receptor complex allows a family of cytokines sharing a common receptor to elicit differential responses by controlling kinetics of downstream signal activation. However, the type I IFN family is quite unique, and therefore other cytokines use different mechanisms, beyond the half‐life of their receptor complexes, to control their signal activation and biological activity potencies. Some cytokines rely on the kinetics of receptor complex formation to define the identity of their signalling and functional profiles. A well‐studied example of this is found in the IL‐4/IL‐13 system. IL‐4 and IL‐13 are two cytokines that play a central role in establishing type II immune responses to fight parasites [24, 58, 59]. Their dysregulation is strongly associated with the development of allergies and asthma, making them an attractive target for pharmacology manipulation [58]. IL‐4 and IL‐13 share surface receptors and signalling programs leading to a strong overlap in their biological profiles [23, 60, 61]. Yet pockets of specificity can be found between these two cytokines that allow them to control different aspects of the immune response [26, 62, 63]. IL‐4 is essential for immunoglobulin class switching and for Th2 differentiation [24, 58, 59], while IL‐13 is the key effector cytokine contributing to airway hypersensitivity responses and expulsion of parasites [64]. IL‐4 engages a type I receptor complex comprised of IL‐4Rα‐γc subunits that is exclusively found in haematopoietic cells [61, 65]. IL‐4 can also bind a type II receptor formed by IL‐4Rα and IL‐13Rα1 subunits, which it shares with IL‐13 [61, 65]. While both cytokines activate STAT6 downstream of the type II receptor, IL‐4 is at least ten times more potent in engaging this signalling pathway than IL‐13 [59, 61, 66]. Paradoxically, in this system, the weaker cytokine – IL‐13 – is the one inducing a more stable receptor complex, raising the question of how the short‐lived complex engaged by IL‐4 promotes activation of STAT6 so efficiently [61, 67, 68, 69, 70].

Works by William E. Paul and others have shown that the kinetics of complex formation by IL‐4 and the surface density of its receptor subunits contribute to the high signalling efficiency exhibited by this cytokine [26, 71]. IL‐4 recruits IL‐4Rα with very high affinity (in the pm range) in a first step, then recruits γc or IL‐13Rα1 to the binary complex with low affinity in a second step. IL‐13 follows an opposite receptor recruiting kinetics, binding IL‐13Rα1 in a first step with moderate affinity (nm range) and then recruiting IL4Rα to the signalling complex in a second step with lower affinity [61, 69, 70]. Due to its high affinity for IL‐4Rα, IL‐4 can initiate the formation of the signalling complex at very low concentration of cytokine, thus ensuring highly efficient signal activation in conditions where γc or IL‐13Rα1 subunits are not limited. IL‐13, on the other hand, requires higher doses to initiate the formation of the signalling complex, but due to its overall stronger stability, this cytokine is more resistant to changes in receptor concentrations [26].

**Fig. 2:**
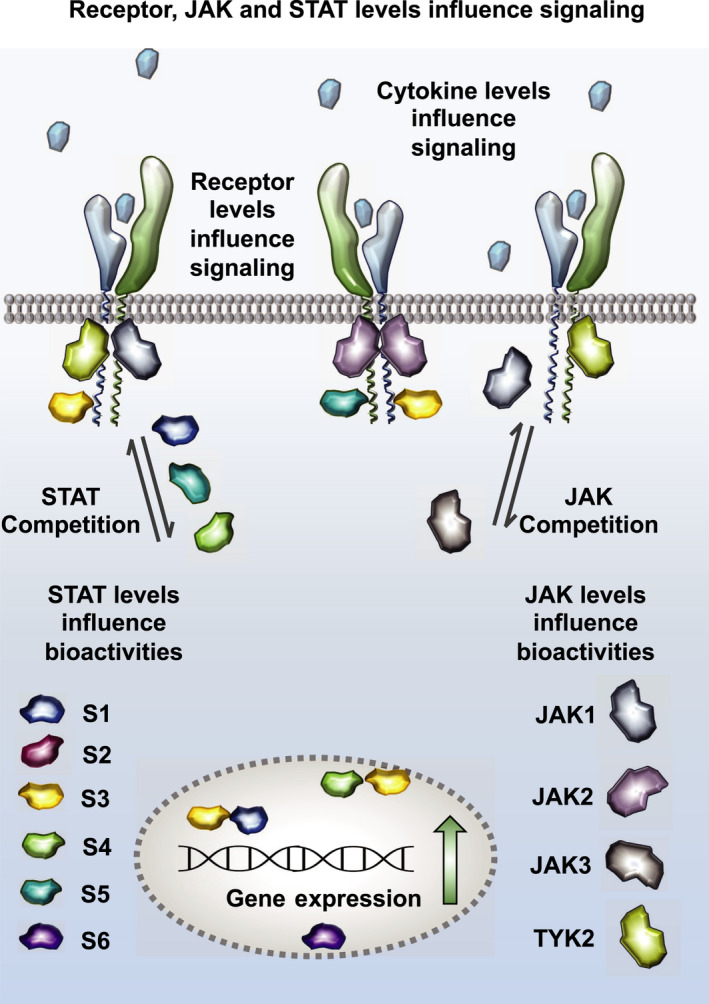
Cellular determinants contributing to cytokine functional pleiotropy. The concentration of cytokine‐receptors, JAKs and STATs molecules influence the nature of the response elicited by any given cytokine. JAKs can compete for binding to their receptors, like for instance in the case of GP130, which ultimately may influence the stability of the cytokine‐receptor complex formed and signalling. While most cytokines activate a dominant STAT to elicit their responses, they are all capable of activating other STATs, which contribute to fine‐tuning their responses. STATs compete with one another for a shared pool of phosphotyrosine in the cytokine‐receptor intracellular domain. Changes in STAT protein levels, due to environmental changes, can thus alter cytokine responses and cause disease.

Another cytokine system where kinetics of complex formation has evolved to define signalling and biological activities is IL‐10 [72]. IL‐10 plays a prominent role in curtailing immune‐mediated inflammation in the context of infection and autoimmunity [9]. Mutations in IL‐10 and its receptors result in early‐onset colitis in humans [73, 74, 75]. IL‐10 is a homo‐dimeric cytokine that engages a receptor comprised of two molecules of IL‐10Rα and two molecules of IL‐10Rβ subunits [72, 76] triggering the activation of the JAK1/TYK2/STAT3 signalling pathway [9]. Interestingly, two viruses, cytomegalovirus (CMV) and Epstein–Barr (EBV), express IL‐10 homologues in their genomes known as viral (v) IL‐10 [77, 78, 79]. These vIL‐10 molecules exhibit very similar sequence and structural homology with human IL‐10, but they only engage the immune‐suppressive arm of the IL‐10 response, thus facilitating virus propagation [77, 78, 79, 80]. vIL‐10 induces strong inhibition of MHC class II expression by cells of the innate immune compartment and blocks T cell proliferation, without promoting the activation of mast cells and the upregulation of MHC class II by B cells [78, 81, 82, 83]. Early studies showed that vIL‐10 binds IL‐10Rα with 1000‐fold lower affinity than human IL‐10, making vIL‐10 extremely sensitive to changes in IL‐10Rα concentration, and explaining its poor potency in cells expressing low IL‐10Rα levels [84, 85]. Strikingly, despite its lower binding affinity, vIL‐10 elicits stronger responses than human IL‐10 in cells where the concentration of IL‐10Rα is not limited [85]. Studies where the binding affinities and complex formation dynamics by human IL‐10 and vIL‐10 were compared shed some light into this functional conundrum. These studies suggested that the lower IL‐10Rα binding affinity exhibited by vIL‐10 could allow it to serially trigger activation of a larger number of receptor complexes than human IL‐10 [78]. The short‐lived complexes engaged by vIL‐10 may allow signal activation without inducing receptor internalization and degradation, thus contributing to more sustained and potent signalling responses [78]. Indeed, similar observations were also made for type I IFNs. IFNα2, which binds IFNAR1 and IFNAR2 subunits with lower affinity than IFNβ, can trigger more potent STAT phosphorylation in cells expressing high levels of IFN receptors [50]. Here again, a serial triggering mechanism was proposed to explain this behaviour. Overall, these studies describe an intricate relationship between ligand‐receptor complex stability, surface receptor densities, signalling and biological outcomes by cytokines, all of which contribute to functional heterogeneity (Figs 1 and 2).

### Manipulating cytokine‐receptor complex stability to fine‐tune signalling responses

The strong inter‐relationship observed between cytokine‐receptor binding affinity/kinetics and signalling and functional potencies suggests that manipulation of cytokine‐receptor binding properties could potentially be exploited for therapeutic purposes. Indeed, in the last ten years, we have seen an explosion of work where many cytokines have been engineered to alter their bioactivity profiles [33, 34]. This has produced new exciting molecular insights into understanding how the binding of a cytokine to its cognate receptor is conveyed inside the cell to activation of specific signalling programs and the induction of unique biological responses.

IL‐2 is a clear example of how manipulation of cytokine‐receptor binding properties via protein engineering has helped to better understand cytokines’ complex biology and to design more efficient and less toxic cytokine therapies [33, 34, 86]. IL‐2 plays a critical role in modulating both immune‐effector activities to fight cancer and inducing immune tolerance to prevent autoimmune disorders [6, 7, 87, 88]. These IL‐2 dual activities have made its biology very complex and its clinical profile difficult to predict. While IL‐2 is currently used in the clinic to treat certain types of cancer [89, 90], the high toxicity associated with its use has prevented its wider application [91, 92]. Decoupling IL‐2 immunostimulatory and immunosuppressive activities is therefore highly desirable. IL‐2 can engage two types of receptors to elicit its immune‐modulatory activities, a high‐affinity hetero‐trimeric receptor comprised of IL‐2Rα, IL‐2Rβ and IL‐2Rγ subunits and an intermediate affinity hetero‐dimeric receptor comprised of IL‐2Rβ and IL‐2Rγ subunits [93]. Both receptors trigger the activation of the JAK1/JAK3/STAT5 signalling pathway [7, 87]. IL‐2 also engages non‐STAT pathways that contribute to fine‐tuning its functional properties [7]. IL‐2 assembles its receptor sequentially, first binding IL‐2Rα with high affinity, and then recruiting IL‐2Rβ and IL‐2Rγ to the complex with lower affinities [11, 94]. In the absence of IL‐2Rα expression, higher doses of IL‐2 are required to form the intermediate affinity receptor – IL‐2Rβ‐IL‐2Rγ – and activate signalling. While IL‐2Rα exclusively binds IL‐2, IL‐2Rβ also serves as a receptor for IL‐15 and IL‐2Rγ is shared with five other cytokines, forming the so called Common γc cytokine family [6, 87]. IL‐2Rα lacks a functional intracellular domain and does not contribute to signalling, but relies on phosphorylation of tyrosines in the intracellular domain of IL‐2Rβ [6, 7]. However, the dynamic nature of IL‐2Rα expression makes this subunit fundamental to defining the cell sensitivity to IL‐2 and therefore IL‐2 responses. IL‐2Rα is constitutively expressed on T reg cells, but not in NK cells or resting effector CD8 T cells [87]. Upon T cell receptor (TCR) stimulation, levels of IL‐2Rα are rapidly induced which heightens the cellular sensitivity to IL‐2 [7, 87]. Extensive characterization of IL‐2 receptor binding dynamics has provided the molecular framework to design IL‐2 variants with altered receptor subunit affinity, potency and target cell specificity [11, 94].

Early engineering studies soon realized that an optimal way to manipulate and reduce IL‐2 cellular targets was to fine‐tune its binding affinity for the IL‐2Rα receptor subunit. These studies generated IL‐2 mutants that bound with higher affinity to IL‐2Rα [95]. These mutants expanded more efficiently in cells expressing high levels of IL‐2Rα, such as T reg cells, and therefore had the potential to promote potent immunotolerance responses [95, 96]. These mutations were later combined with mutations that disrupted IL‐2 binding to either IL‐2Rβ or γc to generate an antagonistic molecule. These IL‐2 antagonists could be used in cancer immunotherapy to repress T reg activities in the tumour [96]. T reg activities could also be prevented by engineered IL‐2 mutants which had reduced binding affinity to IL‐2Rα [97]. These mutants promoted stimulation of CD8 T cells and NK cells, without eliciting T reg cell expansion. As a result of this, these mutants repressed metastasis more efficiently than IL‐2 wildtype and with less toxicity [97]. Later studies showed that IL‐2Rα expressing cells could be efficiently and specifically expanded by IL‐2, when the affinity of this cytokine for IL‐2Rβ was decreased [98, 99]. IL‐2 mutants exhibiting reduced affinity for IL‐2Rβ promoted specific growth of T cells versus NK cells, reflecting the higher IL‐2Rα expression by the former cell type [100]. It was proposed that these mutants could have a better therapeutic index to treat cancers and infection, by inducing lower toxicity as a result of weaker NK activation. However, early clinical trials in HIV infection, advanced melanoma or renal cancer did not show any advantage of using these mutants as compared to wildtype [101, 102], since the high doses required for therapeutic effect nullify their selective T cell growth advantage [103]. In principle, these mutants could also be used to expand T reg cells in autoimmune disease, since these cells express high levels of IL‐2Rα. Indeed, low‐dose IL‐2 therapy is known to preferentially expand T reg cells, again highlighting the high sensitivity to IL‐2 exhibited by this cell subset [104, 105, 106].

Follow‐up studies have continued to devise more sophisticated approaches to manipulate IL‐2 binding to its alpha and beta receptors, generating new IL‐2 variants capable of engaging either the immune‐effector or immune‐suppressive arm of the IL‐2 response to improve anti‐tumour responses or promote tolerance respectively (reviewed in [33, 34, 35]). An example of these new approaches is Neoleukin, a computationally designed protein that interacts exclusively with IL‐2Rβ and the common γc to trigger signalling. Neoleukin possesses superior therapeutic activity when compared to IL‐2 in mouse models of melanoma and colon cancer, with a reduced toxicity [107]. While these approaches have generated interesting new IL‐2 variants with improved therapeutic profiles that are currently in different stages of clinical development, they do not address how the IL‐2 receptor complex half‐life influences signalling potency and bioactivities. Two recent studies have started to provide some molecular insights into this question. Mitra and colleagues showed that by decreasing the affinity of Super‐2, an IL‐2 mutant that binds with high affinity to IL‐2Rβ [108], to the IL‐2Rγ receptor subunit, a partial IL‐2 agonist could be generated, which induced STAT5 Tyr phosphorylation with different amplitudes [109]. The short‐lived complexes formed by these IL‐2 mutants could only trigger partial signalling responses even at saturating doses. Using these mutants, the authors identify differential IL‐2 bioactivity thresholds in different T cell subsets. IL‐2 weak agonists could not support proliferation of naïve T cells but promoted proliferation in pre‐activated T cells [109]. A follow‐up study further confirmed the therapeutic potential of this approach by showing that one of these mutants could lead to the differentiation of CD8 stem‐like cells, driving potent anti‐tumour responses [110].

The observation that different cytokine bioactivities require different levels of signal activation is not unique to IL‐2 and was initially described in the type I IFN system [39]. Type I IFNs can mount potent antiviral responses even at doses that lead to undetectable signal activation. Indeed, most IFN subtypes elicit comparable antiviral responses, despite exhibiting very different receptor binding affinities and complex stabilities [39]. However, the same does not apply for an IFN anti‐proliferative effect, which requires long‐lived receptor complexes and potent signalling responses to be induced [47, 54]. Due to these different requirements for signalling, the two bioactivities can be decoupled by mutants that form short‐lived receptor complexes. Levin et al. [111] proved this by engineering an IFNα2 mutant that bound IFNAR1 with no detectable affinity. This IFN mutant triggered very weak signalling, but led to potent antiviral responses, without exhibiting anti‐proliferative properties [111]. Nevertheless, we still do not understand how changes in complex stabilities generate partial agonism in the cytokine system, and how different signalling amplitudes lead to decoupling of cytokine responses.

The concept of partial agonism is well established in other ligand‐receptor systems such as the GPCR system. Here different ligands promote specific structural alterations in the transmembrane (TM) helices of the engaged receptor, which allows it to recruit and activate differential signalling pathways [112]. However, this model is difficult to reconcile with the cytokine system, where cytokine‐receptors are constitutively associated with JAKs, which in turn phosphorylate Tyr in the intracellular domain of the receptors to recruit STATs and other signalling molecules. Moreover, in this system, partial agonism is achieved by changes in the cytokine‐receptor complex stability, which do not result in changes in receptor binding topology [3]. To gain insight into how cytokine partial agonism is achieved and contributes to functional decoupling, we recently engineered new molecular tools focused on the IL‐6 system to study how cytokine‐receptor complex stability is translated into differential signalling responses [40].

IL‐6 is a very pleiotropic cytokine that acts as a central regulator of the immune response [8, 113]. IL‐6 regulates the balance between Th‐17 and T regulatory (Treg) cells by promoting the differentiation of the former cells and inhibiting the differentiation of the latter, thus promoting inflammation [113]. In addition, IL‐6 exhibits non‐inflammatory activities such as stimulation of muscle regeneration and metabolism regulation [114]. IL‐6 signalling is often found deregulated in human diseases, making this cytokine highly relevant for human health [8, 115]. However, little is known regarding how IL‐6 elicits its pleiotropic activities. IL‐6 engages a hexameric complex comprised of two IL‐6Rα and two GP130 receptor subunits to activate the JAK1/STAT1/STAT3 signalling pathway [116, 117]. The signalling capability of this hexameric complex lays exclusively on GP130 dimerization, since IL‐6Rα lacks an intracellular domain [118]. IL‐6 can also engage in a tetrameric complex (IL6/IL‐6Rα/GP130/GP130) able to trigger signalling [119]. However, the functional relevance of these tetrameric complexes and whether they occur *in vivo* is under debate and requires further studies. To explore how the stability of the IL‐6 receptor complex modulates its signalling responses, we engineered IL‐6 to bind with different affinities to GP130 [40]. Interestingly, our data showed that short‐lived IL‐6/GP130 complexes partially activate signalling but fail to induce the full spectrum of IL‐6 biological responses. Short‐lived IL‐6/GP130 complexes induced biased STAT1 versus STAT3 signalling, with these complexes activating STAT3 but not STAT1 [40]. These observations evoke a kinetic proof‐reading mechanism for cytokine signalling. This model, which has been proposed for the TCR [120] and receptor tyrosine kinases (RTK) systems [121], establishes that changes in ligand‐receptor complex dwell‐times induce phosphorylation of different Tyr pools in the receptors intracellular domains, ultimately recruiting and activating different signalling effectors. However, the cytokine system differs significantly from the TCR and RTK systems. While in these latter systems specific Tyr residues in the receptor intracellular domains engage unique downstream signalling [122, 123], this is not true for cytokine‐receptors where generally Tyr residues play a redundant role, all contributing to engage the full signalling program engaged by a given cytokine [124, 125, 126]. Hence, how can cytokine‐receptor complex dwell‐times be translated into different signalling outputs, when the signalling effectors are competing for a pool of common phosphorylated Tyr residues?

Our data support a “differential saturation model” of cytokine signalling, whereby different levels of active cytokine‐receptor complexes are required to achieve maximum phosphorylation of different STAT molecules. However, further experimental evidence is needed to validate this model. In practice, this model proposes that cytokine‐receptor complex dwell‐times correlate with the number of Tyr phosphorylated in the receptor intracellular domains. Long‐lived complexes produce higher numbers of phosphorylated Tyr than short‐lived complexes. Due to the competitive nature of the binding between STATs for available phospho‐Tyr, STATs binding with low affinity to phosphotyrosine require longer‐lived cytokine‐receptor complexes and higher ligand doses to reach maximal activation. In agreement with this model, previous studies showed that STAT1 and STAT3 bind with different affinities and compete for the same phospho‐Tyr motif in GP130 [127]. Moreover, changes in STAT concentrations have been shown to alter the nature of the responses induced by certain cytokines (Fig. 2). Enhanced STAT1 protein levels, because of IFN gamma treatment, shift the IL‐10 response from STAT3 activation to STAT1 activation [128]. In cells lacking STAT3, IL‐6 switches to STAT1 activation, producing IFNγ‐like responses [129].

This model is not exclusive to the IL‐6 system and appears to be widely used by cytokines to fine‐tune their responses and provide functional diversity. For example, IL‐10 mutants that engage short‐lived complexes act as partial biased agonists, activating more efficiently STAT3 than STAT1 [76, 130]. Conversely IL‐10 variants that formed more stable complexes than wildtype could trigger more potent STAT1 activation, further supporting the competitive nature of the STAT‐pTyr interaction [130]. Similar observations were made for IL‐22, where engineered variants that formed short‐lived complexes promoted STAT3 activation, but not STAT1, exhibiting decoupling of their pleiotropic activities [131]. Our model proposes that STAT affinity to cytokine‐receptor phosphotyrosine motifs controls STAT phosphorylation kinetics and the identity of the gene expression program engaged by cytokines, ultimately ensuring the generation of functional diversity using a limited set of signalling intermediaries. In the future, it would be interesting to investigate whether cytokine‐receptors shared by multiple cytokines, like for instance GP130, common gamma and common beta chains, use this signalling mechanism to differentiate the cytokine bound in the extracellular domains (ECDs) and adapt their signalling responses accordingly. Moreover, since receptor and STAT levels vary across cells and in healthy versus disease conditions, understanding how this ultimately regulates cytokine responses will be crucial to improve our ability to predict and manipulate cytokine behaviour.

### Cytokine‐receptor complex geometry and signalling responses

While the stability of the cytokine‐receptor complex plays a central role in defining cytokine responses, other receptor binding parameters also contribute to fine‐tuning cytokine signalling and responses [132]. Cytokines bind their receptors in a diverse range of molecular architectures and stoichiometries [56, 61, 94, 116, 133, 134, 135, 136, 137, 138, 139, 140, 141]. Yet all of these configurations converge into activation of similar JAK/STAT signalling programs [17], raising the question of whether cytokine signalling only requires receptor proximity to activate JAKs or if particular receptor geometries play instructive roles in determining the degree and nature of receptor activation. To date, this question remains the subject of intensive debate in the field. The literature is full of studies arguing in favour of both models, making it difficult to navigate and draw strong conclusions. Early studies using chimeric cytokine‐receptors, where the ECD of a cytokine receptor was swapped with the ECD of another cytokine receptor, suggested that the topological requirement for signal activation by cytokines are quite lax [142, 143, 144, 145, 146]. In all studies, the chimeric receptor triggered signalling to levels comparable to those induced by the wild‐type receptor. Moreover, forced dimerization of cytokine receptors in non‐canonical topologies also drive potent signalling responses. For instance, replacement of the ECD of GP130‐type receptor complexes (GP130, LIFR, OSM‐R, WSX‐1 and GPL) with the IL‐15/IL‐15Rα‐sushi domain promotes constitutive dimerization and sustained STAT1/STAT3 responses [147]. Similar results were obtained when the c‐jun leucine zipper region was used as the dimerization motif [148]. The lax topological requirements hold true even when the cytokine‐receptor ECD was swapped with the ECD of a tyrosine kinase receptor, such as EGFR and cKit [142]. More recently, we provided further evidence supporting these observations [149]. We generated a large matrix of cytokine‐receptor chimeras where a hundred different cytokine receptor pairs were studied. This study revealed that most combinations tested activated signalling to some extent, again arguing against the requirement of a strong topological constrain for cytokine signal activation [149]. However, although this was true at the population level, there were some specific cytokine‐receptor combinations that did not activate signalling. Modifications in their intracellular domain topologies could rescue signalling by these receptors’ pairs, suggesting that in some instances, receptor binding topology could contribute to cytokine signal activation [149].

In apparent contrast to the conclusions drawn using cytokine‐receptor chimeras, other studies have presented evidence supporting a model where receptor binding topologies critically contribute to define the nature of the signalling activated by cytokine‐receptors. A limitation to these studies is that they were performed mostly in homo‐dimeric cytokine‐receptor systems, for example, Epo, Tpo and GH, raising the question of whether these observations also apply to hetero‐dimeric cytokine‐receptors, the most abundant cytokine‐receptor class. Early studies by the Lodish lab in the Epo system showed using disulfide‐mediated EpoR dimerization that not all EpoR dimers could trigger signalling [150, 151, 152]. Cysteine pairs were introduced in the EpoR TM domain to induce ligand‐independent EpoR dimerization with different topologies and orientations. Only three out of the six EpoR dimer orientations generated activated robust signalling, suggesting that specific TM orientations are required to initiate signalling by Epo [150, 151, 152]. Later studies showed that the interhelical packing of the EpoR TM segment played a central role not only in signalling initiation but also in determining signalling identity [153]. The introduction of asparagine in the EpoR TM generated EpoR mutants that could activate STAT5 to levels comparable to those induced by wild‐type EpoR but failed to engage the Erk1/2 signalling pathway [153].

Further evidence supporting the role of cytokine‐receptor binding topologies in signal activation can be found in studies using chimeric EpoR receptors. In these studies, the EpoR ECD was exchanged with the coiled‐coil dimerization domain of the yeast transcription factor Put3. In the absence of ligand, this domain induces dimerization of the receptor and the activation of signalling [154]. To study how receptor orientation influences signalling, the authors inserted alanine residues in the juxtamembrane region of the EpoR intracellular domain, which is predicted to be helical [155]. Introduction of alanine residues in this region would result in a rotation of the helix by approximately one‐third of a turn, thus altering the orientation of associated JAKs and therefore presumably signalling. Indeed, the insertion of alanines had a differential effect in the downstream signalling activated by the chimeric Epo receptor, with some combinations blocking signalling completely and others leading to biased signalling responses or wild‐type responses [155]. These findings were later confirmed in other homo‐dimeric receptor systems such as Tpo and GP130, suggesting that homo‐dimeric receptors prefer a specific orientation in their intracellular domains to fully engage their signalling program and biological responses [156, 157]. However, not all homo‐dimeric receptors seem to follow this model. Manipulation of the TM domain of the prolactin receptor did not impact its ability to trigger signalling [158]. A further limitation to these studies is that they assumed that the modifications introduced in the TM or juxtamembrane regions had an impact on the topology of the cytokine‐receptor complex without any structural evidence. Thus, at this point, in the absence of direct structural information, these conclusions remain speculative.

Several studies have provided conflicting evidence regarding the role of cytokine‐receptor binding topology and signalling. Studies using Epo mimetic peptides able to trigger EpoR dimerization showed that alternative EpoR binding topologies were compatible with full agonistic activities [137, 159, 160]. These peptides symmetrically dimerized EpoR and triggered potent STAT5 activation and proliferation of Ba/F3 cells. Strikingly, the same authors found that the addition of two bromo groups to the agonistic peptide abolished its signalling capability despite promoting EpoR dimerization [136]. The crystal structure of the EpoR complex formed by this non‐agonistic peptide revealed that the rotational orientation of the dimer formed by the non‐agonistic and agonistic peptides differ by approximately 15 degrees, suggesting that small changes in cytokine‐receptor binding topology could have dramatic consequences in signal activation [136]. A major caveat to these results is that the non‐agonistic peptide bound EpoR with 200‐fold lower affinity than the agonistic peptide, making it difficult to assign the signalling differences to either changes in receptor binding topology or stability. Indeed, a later study showed that the non‐agonistic Epo mimetic peptide could trigger signalling in cells highly sensitive to Epo [161], indicating that further studies are required to understand the contribution that structural and affinity effects play on cytokine functional outcomes.

More recently, a series of studies using Epo and Tpo surrogate ligands have provided new evidence that help to reconcile the two previous conflicted visions – does cytokine‐receptor binding topology instruct signalling output properties? The first of these studies used diabodies against EpoR to homodimerize this receptor in different conformations and determined their impact on downstream signalling [161]. Diabodies are noncovalent dimers of single‐chain Fv (ScFV), where the linker connecting the heavy variable (VH) and light variable (VL) chains has been shortened to only five amino acids [162]. This allows the anti‐parallel dimerization of two diabody chains, forming the active molecule, which has two binding sites. In addition, diabodies are structurally constrained, when compared with full antibodies, thus allowing their structural characterization [162]. Three diabodies were described in this initial study, which enforced dramatically different binding topologies in the EpoR complex. Strikingly, despite these large differences in binding geometry, two out of the three diabodies triggered signalling, with one of them behaving like a full agonist. These results showed for the first time that cytokine‐receptor dimer architectural and spatial constraints compatible with signalling are very liberal. Ligands positioning the receptor ECDs at distances ranging from 30 to 100 Å could promote full agonistic activities [161]. Indeed, this agrees with the wide range of receptor binding topologies and stoichiometries engaged by cytokines to initiate signalling. However, this study also revealed that not all receptor binding topologies could activate signalling, indicating the existence of some structural constrains in the cytokine system. Only one out of three diabodies could trigger full agonistic activities, with the other two promoting partial activities or no activities at all, despite all of them dimerizing EpoR with similar efficiencies [161].

However, this study did not address which topological factors – inter‐receptor distances or rotational angles – had a stronger influence in signalling. A second study focused on addressing this question by using designed ankyrin repeat proteins (DARPins) to create dimeric surrogate ligands with exquisite control of the relative orientation and spacing of the receptors’ ECDs in a dimeric complex [163]. This study revealed that both parameters contribute to signalling efficiency; however, distortion of the EpoR rotational angle seems to promote stronger changes in signalling than modification of inter‐receptor distances. Changes in EpoR distances have very little effect in signalling until a certain threshold is reached, upon which signalling decreases abruptly [163]. This is not exclusive to the Epo system and similar observations were obtained for thrombopoietin (Tpo) [163]. Diabodies targeting the thrombopoietin receptor exhibited very similar functional profiles to those designed for the Epo system, suggesting that these structural principles are common to all homo‐dimeric receptors [164]. However, whether these structural principles hold true in the larger hetero‐dimeric cytokine receptor family remains an open question.

Overall, these studies highlight that cytokines can accommodate a large range of receptor binding topologies to initiate signalling, but that protein engineering can be used to push these structural boundaries to fine‐tune cytokine responses. Yet, how these extreme receptor binding geometries imposed by cytokine surrogate ligands contribute to alter downstream signalling remains an open question. The most accepted hypothesis is that they result in mechanical distortions that promote inefficient activation of associated JAK kinases. This in turn leads to differential phosphorylation of Tyr in the receptor intracellular domains, which ultimately promote the differential responses observed. However, without structural evidence and more detailed characterization of phosphotyrosine profiles engaged by these altered receptor complexes, this remains highly speculative. Nonetheless, it appears that both changes in receptor complex stability and/or binding geometries converge into the same functional principle to manipulate signalling, that is, manipulation of phosphotyrosine numbers in the receptor intracellular domain, agreeing with our differential saturation model for cytokine signalling. Thus, it is tempting to speculate that any cytokine or receptor manipulation that alters the number of Tyr residues phosphorylated in the receptor intracellular domain can be exploited to decouple cytokine functional pleiotropy.

## Cytokine‐receptor – JAK interaction and signalling

Janus kinases translate extracellular cytokine‐receptor binding information into phosphorylation of Tyr residues in the receptor intracellular domains and into activation of specific STATs programs, ultimately controlling the nature and potency of the cytokine response. However, despite their vital role in cytokine biology, we still lack a molecular understanding of how signalling is initiated by JAKs upon cytokine‐receptor complex formation. Many excellent reviews addressing this aspect of JAK biology can be found in the literature and therefore we will not further expand on this topic [5, 13, 17, 18, 165, 166, 167]. Here, we will focus on two non‐canonical JAK activities, going beyond its ability to phosphorylate Tyr, which critically contributes to fine‐tune cytokine signalling: (a) JAKs role in maintaining cytokine‐receptor density in the surface of responsive cells and (b) JAKs contribution to the cytokine‐receptor complex stability.

### JAKs and cytokine‐receptor surface density

Cytokine‐receptor surface density critically contributes to define cells’ sensitivity to cytokines. Immune cells manipulate their cytokine‐receptors expression to adapt their responses to their microenvironment. While changes in receptor density can be manipulated epigenetically, often JAKs can play a more immediate and direct role in modulating cytokine‐receptors surface expression [168, 169, 170]. Cytokine‐receptors bind JAKs via two membrane proximal regions in their intracellular domain, known as Box1 and Box2. Box1 is a proline‐rich segment, while Box2 is downstream of Box1, and comprises a hydrophobic segment of approximately 20‐40 amino acids. JAKs bind these two regions via their FERM‐SH2 domain [171]. The elucidation of FERM–SH2 structures from three of the four JAK family members has provided us with a detailed molecular understanding of how JAKs bind their receptors [172, 173, 174]. These structures have revealed key binding determinants that allow JAKs to specifically recognize certain cytokine‐receptors but not others. One example is found in the positioning of the F2‐a3 helix in JAK1, TYK2 and JAK2 [172, 173, 174]. JAK2 presents a rotation of this helix, which allows the formation of a salt bridge between Arg232 and Glu176, closing off the hydrophobic pocket that it is used by JAK1 and TYK2 to bind the receptor [172, 173, 174]. Interestingly, some cytokine‐receptors, including GP130, GHR, G‐CSFR and EPOR, appear to bind more than one JAK protein. An analysis of their Box1/2 domain revealed a different conserved Box1 PxP motif, which was critical for JAK binding. It is possible that the PxP motif has evolved to bind more than one JAK member, to ensure the functionality of those specific cytokine‐receptor systems [171]. However, whether the identity of the JAK‐receptor dimer contributes to signalling specificity or it plays a redundant role is not currently known.

Beyond being critical to initiate signalling, JAKs also play a chaperone role regulating surface expression of some cytokine‐receptors. This JAK role is independent of their kinase activity. Early work on the OSM system showed that OSM‐R levels directly correlate with JAK1, JAK2 or TYK2 expression [169]. This JAK chaperone effect required the interaction of JAKs with the OSM‐R but not JAK tyrosine kinase activity [169]. This work revealed the existence of three dileucine‐like motifs within the interbox1/2 region of the OSM‐R that contributed to poor surface receptor expression in the absence of JAKs [169]. The exact molecular mechanism by which these motifs regulate receptor expression is not currently known, but a possibility is that these motifs impede the correct folding of the receptor, due to their high hydrophobicity, unless masked by JAKs. These properties are not exclusive to the OSM‐R system and have been described for other cytokine‐receptors. JAK2 plays a central role in the surface expression of homo‐dimeric cytokine‐receptors, that is, EpoR, GH‐R and Tpo‐R [168, 175, 176]. It is believed that JAK2 contributes to the correct folding of these receptors in the endoplasmic reticulum (ER) [168]. In the absence of JAK2, the receptors do not reach the surface and are degraded via lysosomal or proteasome pathways [175, 176]. TYK2 also critically contributes to modulate levels of IFNAR1 and thus regulates the IFN responses. In the absence of TYK2, IFNAR1 reaches the cell surface, but it is rapidly internalized into perinuclear endosomal compartments [170]. TYK2 stabilizes IFNAR1 in the cell surface profoundly increasing its expression and the sensitivity of cells to type I IFNs.

Overall, these data show that cells can quickly and transiently regulate their sensitivities to cytokines by modulating their JAK expression levels as highlighted in Fig. 2. This interdependence between JAKs and cytokine‐receptors surface expression ensures that only receptors binding JAKs can interact with cytokines, guaranteeing that every cytokine‐receptor binding event can be translated into signalling. In the absence of this quality control mechanism, cytokine‐receptors not bound to JAKs could act as dominant negatives, behaving as cytokine ‘sinks’ and preventing effective responses [177]. Indeed, there is evidence supporting competition between cytokine‐receptors for limiting JAKs, highlighting the biological significance of this JAK chaperone role [178, 179].

### JAKs and cytokine‐receptor complex stability

Early work in the Type I IFN system showed that certain mutations in TYK2, focused on its kinase and pseudo kinase domains, influenced the affinity of IFNα2 for IFNAR1 without affecting IFNAR1 surface expression levels, suggesting an inside‐out crosstalk between JAKs and receptors [180]. Another example of this inside‐out communication between receptors and cytokines is found in Usp18. Usp18 is an interferon inducible protein which plays a central role in defining cell sensitivity to type I IFNs [181, 182, 183, 184, 185]. Upon IFN stimulation, cells express high levels of Usp18, which limits their responses to further IFN stimulation [181, 182, 183, 184, 185]. Interestingly, this effect holds true when low receptor binding affinity IFN subtypes, like IFNα2, are used, but not when cells are stimulated with high receptor binding affinity IFNs such as IFNβ [181]. Recent studies showed that Usp18 binds the intracellular domain of IFNAR2 and decreases the affinity of this receptor subunit for IFNα2, without affecting its surface levels [181, 186]. The exact molecular mechanism by which Usp18 hinders IFNα2 binding to IFNAR2 are not currently clear, but the most accepted model is that Usp18 influences JAKs, leading to an inefficient complex formation and poor signalling.

These examples suggest a communication between the intracellular signalling‐mediating and the extracellular cytokine‐binding motifs of cytokine‐receptors which ultimately define the overall stability of the receptor complex formed and the identity of the responses elicited by any given cytokine. Recent studies have started to shed some light onto how this inside‐out communication influences cytokine binding and activities. These studies highlight a new role for the pseudo kinase domain of JAKs, beyond its previously described function in regulating the kinase domain activity. JAKs pseudokinase domains interact with each other in the context of the cytokine‐receptor dimer contributing to overall complex stability [187, 188]. Indeed, this model provides molecular insights into the constitutive activities exhibited by some oncogenic JAK mutants, notably somatic mutations such as JAK2 Val617Phe which causes the Philadelphia chromosome–negative myeloproliferative neoplasms (Ph– MPNs) [189]. These JAK2 mutations act by altering and strengthening the intermolecular interactions involving the PK‐PK dimerization interface, resulting in stabilization of receptor‐JAK dimers and driving signalling in the absence of ligand [188]. These studies have revealed an inside‐out communication that allows signalling components interacting with the cytokine‐receptor intracellular domain to regulate the affinity and stability of the cytokine‐receptor complex formed, ultimately impacting signal activation and responses. However, many questions remain: Is this specific to JAK2 or does it also apply to other JAKs? Do specific JAK pairs produce more stable interactions? How is signalling ultimately affected by this JAK‐JAK interaction? Are there other signalling proteins, beyond Usp18, that modulate this process? A deeper understanding of the molecular and structural bases allowing JAKs to trans‐activate each other to initiate signalling will open the door to manipulation of cytokine activities with small molecules or surrogate cytokines engineered to disturb and interfere with this JAK–JAK binding interface in health and disease.

## Cytokine‐receptor endosomal traffic and signalling

**Fig. 3:**
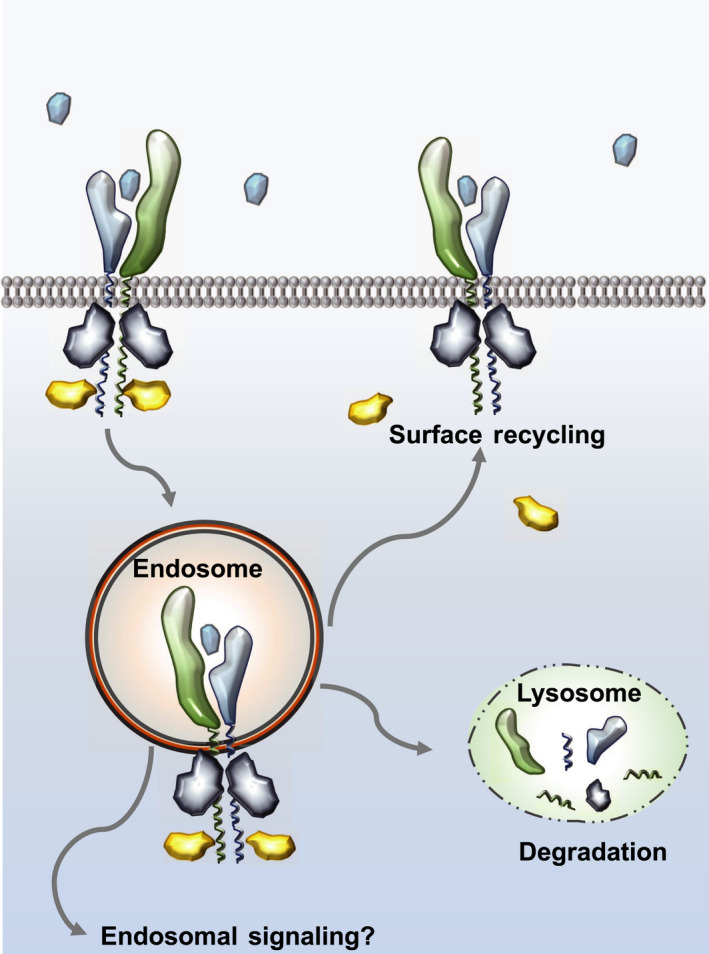
The endosomal compartment contributes to cytokine signalling. In recent years, it has become clear that the endosomal compartment plays a critical role in regulating cytokine signalling and activities. The cytokine‐receptor complex traffics to the endosomal compartment shortly after being formed, where it continues signalling. The endosomal pathway followed by the complex depends on the cytokine system studied and on the stability of the receptor complex formed. Short‐lived complexes tend to dissociate at the acidic pH found in endosomes, and their receptor subunits can follow a recycling route back to the plasma membrane or continue to the lysosome compartment for degradation depending on whether they are ubiquitinated or not. Long‐lived complexes, on the other hand, survive the acidic endosomal pH and continue trafficking through the endosomal compartment increasing their chances of finding additional signalling molecules that contribute to fine‐tuning their responses.

The cytokine‐receptor complex undergoes internalization following cytokine stimulation [37]. The specific endosomal route followed by a cytokine‐receptor complex varies from cytokine to cytokine and can lead to its surface recycling or degradation as illustrated in Fig. 3 [190, 191, 192]. While initially considered an intrinsic mechanism to terminate cytokine responses, now it is clear that the endosomal compartment functions as a signalling hub that contributes to fine‐tuning of cytokine activities [193]. However, several factors have limited our ability to decipher how the endosomal compartment fine‐tunes cytokine signalling and responses. On the one hand, expression of cytokine‐receptors is exquisitely regulated by cells, with receptor copy numbers ranging from hundreds to a few thousand. For instance, both IL‐4Rα and IFNAR1 cell surface levels, receptor subunits for IL‐4 and IFN, respectively, are found to be in the low hundreds [194, 195, 196, 197]. This has precluded us from studying endogenous cytokine‐receptors in live cells and forced us to resort to over‐expression studies that may not reflect the real biology of these receptors. On the other hand, as described in the previous chapter, expression of some cytokine‐receptor subunits is strongly linked to their association with JAKs, further contributing to the difficulty of studying their dynamics via over‐expression approaches [14]. In this context, JAKs expression levels become the limiting factor, resulting in accumulation of cytokine‐receptors in the ER and Golgi compartments. Despite these limitations, reports describing the critical role that the endosomal compartment plays in defining cytokine responses have been steadily appearing over the last 20 years.

Early work on the granulocyte colony‐stimulating factor (G‐CSF) receptor system showed that the endosomal route followed by this receptor critically contributes to its signalling and biological activities profile [198, 199]. G‐CSF is an important immuno‐regulatory cytokine that mobilizes granulocytes from the bone marrow and regulates the survival and differentiation of neutrophils [200]. It does this by activating STAT5 and Akt downstream of the G‐CSFR upon cytokine binding [200]. Interestingly, lack of receptor internalization, resulting from mutations on G‐CSFR that truncate its ICD, promotes sustained G‐CSF signalling and results in myeloid leukaemia and severe neutropenia, confirming the important role that receptor internalization plays in cytokine signalling termination [198]. However, this is not the only function of the endosomal compartment in G‐CSF signalling. G‐CSFR mutants that cannot traffic to the lysosome for degradation get accumulated in early endosomes where they continue activating STAT5 but not Akt, producing defective responses [199]. These observations suggest that the endosomal compartment can act as a signalling hub where active cytokine‐receptors can encounter specific signalling molecules that contribute to define the nature of the response elicited. Importantly, this is not unique to the cytokine system, with similar behaviour having been reported for receptors of the tyrosine kinase family such as EGFR and FLT3 [201, 202].

Another cytokine system where differential endosomal traffic has been shown to critically regulate responses is the type I IFN system. Type I IFNs bind two receptor subunits – IFNAR1 and IFNAR2 – to initiate signalling. Interestingly, the two subunits have very different intracellular traffic in both homeostatic conditions and in response to IFN stimulation [47, 48, 203, 204]. This has been proposed to contribute to the differential activities exhibited by the different IFN subtypes [39]. In response to IFN stimulation, IFNAR1 is phosphorylated on Ser535, which initiates a signalling cascade that terminates with the ubiquitination and degradation of this receptor subunit [205]. This process is initiated by all IFN subtypes independent of their binding affinity or activity profile. IFNAR2, on the other hand, is not ubiquitinated upon IFN stimulation and can undergo recycling or degradation depending on the IFN subtype used [47, 48, 203, 204]. IFN subtypes binding IFNAR1 with high affinity form stable complexes that survive the acidic pH found in endosomes and traffic to the lysosome compartment where both IFNAR1 and IFNAR2 are degraded [206, 207]. IFN subtypes binding their receptors with low affinity, on the other hand, form short‐lived complexes that do not survive the acidic pH found in endosomes. In this context, IFNAR2 dissociates from IFNAR1 in endosomes and recycles back to the cell surface [206, 207]. Immunoprecipitation studies support this model and show that ternary complexes formed by high‐affinity IFN subtypes remain intact long after STAT activation [208]. This suggests that IFN signalling continues as the complex traffics through the endosomal compartment. This signalling mechanism would exclusively favour long‐lived IFN‐receptor complexes, which could contribute to their more potent responses. In agreement with this, it was shown that JAK/STAT molecules translocate to the endosomal compartment upon IFN stimulation [209].

To understand the exact role that the endosomal compartment plays on cytokine signalling and responses, first we need to be able to systematically manipulate and/or block the internalization of any given cytokine‐receptor, without affecting the overall intracellular traffic of the cell system used. However, this has been a challenging problem. Cytokine‐receptor trafficking can be blocked via small molecule inhibitors that target the clathrin pathway, by genetically silencing clathrin via siRNA or by the over‐expression of dominant negative (DN) forms of dynamin [190, 210]. However, when inhibition of intracellular trafficking is efficient, high toxicity occurs. Conversely, when the blockage of intracellular trafficking is poor, you get a minimal effect on cytokine responses. Despite these limitations, several studies have described a complex network of trafficking proteins that contribute to cytokine signalling. Studies where IFN receptor traffic was blocked by silencing clathrin or by using DN dynamin showed that intracellular trafficking contributes to enhance STAT phosphorylation and antiviral responses upon IFN stimulation [190, 210]. Interestingly, when only IFNAR1 degradation is blocked, without affecting its internalization, stronger IFN responses are observed, again suggesting that active IFN complexes accumulate in the endosomal compartment where they continue signalling [205]. Indeed, the endosomal sorting machinery appears to play a critical role in defining JAK/STAT signalling by IFNs. Signal‐transducing adaptor molecule (STAM2), a member of the endosomal sorting complex required for transport complex (ESCRT‐0) [211], interacts with IFNAR1 and TYK2 in the plasma membrane, blocking the activation of the latter in resting conditions [212]. Upon IFNα2 stimulation, the IFN‐receptor complex translocates to early endosomes rich in PI(3)P, where hepatocyte growth factor‐regulated tyrosine kinase substrate (Hrs) binds to STAM2, blocking its inhibitory effects and promoting IFN endosomal signalling [212]. Long‐lived IFN‐receptor complexes formed by high receptor binding affinity IFN subtype, like IFNβ, traffic to different endosomal compartments where signalling occurs independently of Hrs‐STAM2 interaction [212]. Hrs appears to have a broader role in regulating cytokine signalling beyond type I IFNs. Hrs can control IL‐2Rβ and IL‐4Rα signalling by directing their endosomal sorting towards the degradation pathway [213, 214]. In addition to the ESCRT complex, the retromer complex, which controls both retrograde transport of endosomal proteins to the trans‐Golgi network (TGN) and the recycling of endosomal cargos to the plasma membrane [215], have been shown to regulate the recycling of IFNAR2 upon IFN stimulation and therefore IFN signalling [216]. In addition, IL‐6Rα and GP130 have been shown to be internalized independently of IL‐6 in a clathrin‐ and dynamin‐dependent process. Interestingly, IL‐6 enhances the recycling of these complexes back to the cell surface which identifies an alternate function of IL‐6 beyond activation of signalling [217]. These studies paint a picture depicting the endosomal compartment as a central signalling hub that controls the duration of the cytokine response and contributes to diversifying cytokine signalling programs in order to fine‐tune cytokine activities as illustrated in Fig. 3. Endosomal trafficking has been shown to be critical for erythropoietin, IL‐6, IL‐13/IL‐4 and many other cytokines [218, 219, 220, 221, 222, 223]. However, we still lack a clear understanding of the endosomal route followed by most cytokine‐receptor complexes, how the stability of the receptor complex influences endosomal traffic and the identity of the signalling programs activated by cytokines in endosomes and their contribution to cytokine responses.

Recent advances in super‐resolution microscopy and single‐particle techniques have started to provide a window into how cytokine‐receptor complexes are formed within the cell membrane, and the ability to track their intracellular traffic in living cells [224]. Using these technologies, the membrane dynamics of IFNAR1 and IFNAR2 receptors could be elucidated, showing that in the absence of IFN, IFNAR1 and IFNAR2 can have two diffusion regimes, one fast and one slow, which could indicate the localization of the receptors in different membrane domains [225]. Upon IFN stimulation, the diffusion constants of the two receptors decrease, as a result of complex formation. Indeed, it is now clear that most cytokine receptors diffuse freely in the surface of responsive cells, and upon cytokine stimulation, they are recruited to the complex to initiate signalling [40, 41, 130, 188, 226, 227, 228, 229]. However, whether signalling is initiated in the plasma membrane or in endosomes is a matter of debate. The canonical cytokine signalling paradigm stipulates that upon cytokine binding, receptors are brought into close proximity in the cell membrane, where JAKs are trans‐activated and signalling is initiated. However, this paradigm was recently challenged. Studies in the IL‐4 system showed that IL‐4 binds with very low affinity to IL‐2Rγ for efficient dimerization at endogenous plasma membrane expression levels [230]. This suggests that an additional mechanism to increase the overall stability of the ligand‐receptor complex could be used by cytokines to ensure robust signalling [230, 231]. Receptor–receptor and JAK–JAK interactions, as described above, contribute to enhance cytokine‐receptor complex stability. However, recent studies suggest that the endosomal compartment also plays a critical role in modulating cytokine‐receptor complex half‐life [230, 231].

The smaller surface area found in endosomes allows this compartment to elicit a subcellular concentration, resulting in higher cytokine‐receptor densities than those found at the plasma membrane [227]. For IL‐4, it was shown that this concentration step critically contributes to its efficient receptor dimerization [230, 231]. We reported similar observations for IL‐13. Using a series of IL‐13 variants engineered to bind their receptor with a wide range of affinities, we could show that cytokine signalling is quite resistant to changes in complex stabilities. Increases in IL‐13 binding affinity for IL‐13Rα1 enhanced IL‐13 signalling only until the half‐life of the receptor complex formed exceeded the rate of endocytosis. After this, further increases in complex stability did not impact signalling potency by IL‐13 [227]. Similar observations have been made in other cytokine systems, for example, IL‐2, IL‐10 and IFN, where mutants that elicit non‐detectable dimerization of surface receptor complexes trigger potent signalling responses [130, 229, 232]. Overall, these studies point to a new role of the endosomal compartment in stabilizing short‐lived cytokine‐receptor complexes to increase signalling efficiency. Whether signalling is initiated at the plasma membrane or exclusively in early endosomes however remains a matter of debate.

We recently provided new evidence in the IL‐6 system that supports a complex role of the endosomal compartment in defining cytokine signalling signatures [40]. IL‐6 is a critical immuno‐modulatory cytokine that regulates the inflammatory response by engaging a hexameric receptor complex comprised of two IL‐6Rα and two GP130 receptor subunits [8, 113]. The signalling capability of this hexameric complex lays exclusively on GP130 dimerization, since IL‐6Rα lacks an intracellular domain [113, 116]. Upon stimulation, IL‐6 triggers the activation of the JAK1/STAT1/STAT3 signalling pathway to elicit its pleiotropic activities. Two different mechanisms of signal activation have been described for IL‐6: the classical mechanism of signal activation, which involves the binding of IL‐6 to a membrane‐bound form of IL‐6Rα, and the non‐classical or trans‐signalling mechanism of signal activation, which involves the binding of IL‐6 to a soluble form of IL‐6Rα and allows IL‐6 to act in every cell in the body [114, 233]. To investigate how IL‐6 pleiotropic responses were modulated by the stability of the receptor complex engaged, we engineered a series of IL‐6 variants binding GP130 with different affinities. These variants could trigger differential signalling responses in the absence of IL‐6Rα expression. Interestingly, we found that the IL‐6 variants promoted biased STAT1 versus STAT3 signalling, with variants engaging short‐lived GP130 complexes activating STAT3 more strongly than STAT1. Moreover, low affinity IL‐6 variants failed to promote GP130 internalization and accumulated in the endosomal compartment. We could show that STAT1 and STAT3 compete for phospho‐Tyr in the intracellular domain of GP130, with STAT3 binding with higher affinity than STAT1. These data agree with a kinetic discrimination mechanism of signalling, where STATs binding with low affinity to phosphotyrosine require longer‐lived cytokine‐receptor complexes and higher ligand doses to reach maximal activation [40]. Overall, our study supported a discriminatory role for the endosomal compartment in defining signalling by IL‐6. Short‐lived GP130 complexes that fail to traffic to endosomes activated STAT3 in the cell surface but fail to phosphorylate STAT1, which required the stabilization of the short‐lived complex in endosomes. This stabilization provided these complexes with the necessary extra time to activate secondary pathways that engage the receptor with low affinity. Further studies are required to fully elucidate the role of the endosomal compartment in cytokine responses, and whether the intracellular localization of STATs influences their availability to long‐ and short‐lived cytokine‐receptor complexes.

## STATs levels and functional pleiotropy

The identity of the cytokine‐receptor complex and its stability critically contribute to determine signalling and biological responses induced by any given cytokine [17, 32]. However, ultimately STATs are the factors that translate receptor binding properties into specific gene expression programs that inform cells about the nature of the responses required [16]. Generally, all cytokines activate a dominant STAT that defines their activities, for example, IL‐6 activates STAT3 [234], IL‐2 activates STAT5 [7], IFN activates STAT1 [16], etc. However, in most cases, cytokines promote the activation of secondary STAT pathways [7, 19]. How these non‐dominant STATs contribute to shape cytokine responses is not well understood. Early studies showed that phosphotyrosine in the receptor intracellular domain appears to play a redundant role in STAT activation [125, 126, 235, 236, 237, 238]. In most systems studied, mutation of individual Tyr residues to Phe to block their phosphorylation did not affect signalling by cytokines unless all Tyr were mutated to Phe at the same time. These observations suggested that any given STAT can bind to any phosphotyrosine in the cytokine‐receptor intracellular domain to initiate signalling. This is clearly observed in the type I IFN system. IFNs can promote the phosphorylation of all STATs present in the responsive cell. Individual mutation of intracellular Tyr on IFNAR2 to Phe did not impact the ability of IFN to phosphorylate all STATs molecules [126, 235, 236]. Mutation of all intracellular Tyr resulted in complete block of IFN‐induced STAT activation, suggesting that STATs compete for phosphotyrosine in the IFNAR2 intracellular domains [126, 235, 236]. These studies implied that the overall concentration of STATs in each cell could dramatically affect the identity of the IFN response elicited. In a later study, we showed that IFN engages different ratios of STAT activation in different immune cell subsets that could contribute to its pleiotropic activities [56].

The competitive nature of STAT binding for intracellular phosphotyrosine is clearly observed between STAT1 and STAT3. Expression of both STATs are regulated by their own activation, with cytokines activating STAT1 leading to enhanced STAT1 expression and cytokines activating STAT3 leading to increases in STAT3 protein levels. Thus, changes in STAT1 and STAT3 protein concentrations ultimately affect cell responses to cytokines. For example, in inflammatory environments, which are rich in IFNγ, cells tend to express higher STAT1 levels, resulting from IFNγ‐induced STAT1 activation [239]. In this context, IL‐10, an anti‐inflammatory cytokine, activates STAT1 rather than STAT3, and contributes to worsening inflammation rather than preventing it [239]. Another example where STAT competition for phosphotyrosine governs the nature of the response elicited can be found in the IL‐6 and IL‐27 system [8, 240]. IL‐6 and IL‐27 activate comparable signalling programs, that is, STAT1 and STAT3, but elicit very different immune responses. IL‐27 is a hetero‐dimeric cytokine comprised of p28 and EBI3 subunits, which binds GP130 and IL‐27Rα in the surface of responsive cells to activate STAT1 and STAT3 transcription factors [240]. IL‐27 can elicit both pro‐ and anti‐inflammatory activities, but its anti‐inflammatory properties seem to be dominant [240]. IL‐6, on the other hand, is a potent pro‐inflammatory cytokine that forms a hexameric complex comprised of two GP130 and two IL‐6Rα receptor subunits to activate STAT1 and STAT3 transcription factors [8]. We and others have shown that the differential activities elicited by these two cytokines arise from the different STAT1 and STAT3 phosphorylation kinetics that they elicit [41, 240, 241]. While both IL‐27 and IL‐6 promotes phosphorylation of STAT3 with similar kinetics, only IL‐27 induces sustained STAT1 phosphorylation. This is achieved by a high‐affinity STAT1/IL‐27Rα interaction site centred around Tyr613 on IL‐27Rα [41]. While phosphotyrosine residues in GP130 can bind both STATs, STAT3 binds with significantly higher affinity than STAT1. This in turn results in sustained STAT3 phosphorylation kinetics and short STAT1 phosphorylation kinetics upon IL‐6 stimulation. The distinct STAT1 and STAT3 kinetic profiles induced by IL‐6 and IL‐27 result in decoupling of their gene expression programs [41]. Both IL‐6 and IL‐27 promote the induction of a shared group of genes that depend on GP130‐STAT3 activation. However, the more sustained STAT1 activation by IL‐27 leads to the regulation of an exclusive group of genes by this cytokine, which contribute to its unique biological profile [41]. We found that IRF1, which is induced very quickly (1 h) upon IL‐27 stimulation and depends on sustained STAT1 phosphorylation for its continued expression, was required for the unique gene program induced by IL‐27 [41]. IRF1 has been shown to synergize with STAT1 and previous studies showed that IL‐27 and IFNγ but not IL‐6 stimulation led to upregulation of this gene in hepatocytes [242]. These results suggest that STAT activation kinetics critically influence the expression of accessory transcription factors, such as IRF1, that act in synergy with STATs to define the nature of the gene program elicited.

The tight coupling of one receptor subunit within the cytokine‐receptor complex to one STAT found in the IL‐27 system is rather unusual for cytokines [243]. Generally, one of the receptor subunits within the complex takes a dominant role driving signalling by carrying most Tyr residues susceptible to being phosphorylated [235, 244]. This generates competition between STAT molecules for binding to those shared phosphotyrosine and therefore different kinetics of phosphorylation between high‐affinity and low‐affinity STATs as described for IL‐6 [245]. This basic model is very efficient and allows for a coordinated signalling wave between positive and negative feedback regulators contributing to cytokine signalling. However, as described above, the system presents its limitations. STAT competition for the same pool of phosphotyrosine makes the system very sensitive to changes in STAT concentration, which could explain why cytokines are so often associated with disease [129]. Interestingly, IL‐27 appears to have evolved away from this general model, allowing this cytokine to elicit robust responses in dynamic immune environments. These data fully support our differential saturation model for cytokine signalling. STAT affinity to specific cytokine‐receptor phosphotyrosine motifs controls STAT phosphorylation kinetics and the identity of the gene expression program engaged by cytokines, ultimately ensuring the generation of functional diversity through the use of a limited set of signalling intermediaries. Since receptor and STAT levels vary across cells and in healthy versus disease conditions, understanding how this ultimately regulates cytokine responses will be crucial to improve our ability to predict and manipulate cytokine behaviour.

## Conclusions

The cytokine field has advanced dramatically since the first cytokine, type I IFN, was discovered in 1957 by Isaacs and Lindenmann [246]. Since then, more than 30 cytokines have been identified, together with their receptors and JAK/STAT signalling pathways engaged [17, 32, 247]. We have gained a deeper insight into the biological processes controlled by cytokines in health and disease, and cytokine therapies, particularly those focused on neutralizing cytokine activities, have become a reality and improve the lives of millions of people world‐wide [247, 248]. However, there are still many outstanding questions that have prevented us from utilizing the full potential of cytokines in the clinic. One of these questions pertains to how cytokines exert non‐redundant activities despite sharing key components of their signalling pathways. An example that better exemplifies this question is found in IL‐6 and IL‐10 cytokines. These two cytokines activate comparable STAT3 levels, yet IL‐6 elicits pro‐inflammatory activities and IL‐10 is the prototypic anti‐inflammatory cytokine [17, 36, 249]. We and others have started to show that in addition to the identity of the STAT molecules engaged by a cytokine, the amplitude of activation also contributes to define the nature of the response. Engineered cytokine variants that elicit partial agonistic responses can decouple functional pleiotropy and thus elicit more specific responses and diminish systemic toxicity [33]. But the question remains as to how different levels of STAT activation promote unique gene expression programs and decouple biological responses. Several studies have shown now that not all cytokine‐induced genes sense changes in STAT activation in the same manner [3, 40, 76, 110, 111, 130, 131, 250]. While induction of some genes is saturated at low STAT activation levels, other genes require high levels of STAT activation to be induced. This generates a gradient of gene expression sensitivity that is exploited by cytokines to promote unique gene expression programs, despite sharing signalling components [40, 111]. Moreover, any environmental stress that changes the levels of receptors, JAK or STAT can significantly alter the response of a given cell to cytokines. However, what differentiates robust from tuneable genes is not known, which has limited our ability to tailor cytokine activities with protein engineering. Moreover, we lack a clear understanding of the role of individual genes in specific cytokine bioactivities. As more cytokine partial agonists are generated and cytokine activities decouple, we will start to comprehend how cytokine signalling leads to specific gene expression programs and pleiotropic activities.

Cytokines can engage accessory signalling pathways, other than the JAK/STAT pathway, that are believed to contribute to fine‐tuning their responses [7, 19]. For instance, in addition to STAT5, IL‐2 can activate the Ras/Erk1/2 pathway and the mTORC1 pathway, with the latter controlling IL‐2 metabolic and transcriptional gene programs, thus shaping T cell proteomes to control energy‐generating metabolic processes [7]. Similarly, Type I IFNs were reported to engage the mTORC1 pathway to regulate protein translation, which critically impacted IFN antiviral activities [19]. However, a recent study could not reproduce these observations [251]. More systematic studies characterizing cytokines entire signalosome in primary cells are needed to better understand how non‐STAT pathways contribute to cytokine responses. We recently combined high‐throughput multiparametric phospho‐flow cytometry with unbiased phosphoproteomic studies to obtain a full picture of the IL‐6 signalosome in human T cells. This study uncovered a novel role of the nuclear compartment in defining IL‐6 responses, via regulation of CDK8 localization [252]. CDK8 regulated STAT3 chromatin binding dwell‐time and transcriptional activities, which could have important implications in STAT3 mediated diseases and inflammation. CDK8 and STAT3 interacted in the nucleus upon IL‐6 stimulation, leading to STAT3 Serine phosphorylation [252]. Inhibition of CDK8 resulted in a more sustained STAT3 Tyrosine phosphorylation and nuclear retention upon IL‐6 stimulation, which led to a global increase in STAT3 chromatin binding intensity at all STAT3 target sites and augmented Th‐17 differentiation [252]. However, CDK8 plays a positive role in regulating STAT1 activities in macrophages, highlighting the complex roles that non‐STAT signalling pathways play in modulating and controlling cytokine responses [253].

New technological advances allowing the monitoring of cytokine‐receptor complexes in real time within the plasma membrane as well as in their trafficking through endosomal compartments will enable us to better understand how these complexes initiate and diversify signalling. Moreover, a global characterization of cytokine signalling signatures, as well as a clear understanding of how STAT activation leads to unique gene expression programs will help us to unravel how cytokines elicit their pleiotropic responses. Combining these advances with protein engineering to precisely manipulate cytokine properties will enable us to probe cytokine systems in a much more defined manner. Overall, a profound understanding of how the binding of cytokines to their receptor complexes is transmitted intracellularly to induce unique signalling and gene expression programs, and a plethora of biological responses will enable us to design more effective cytokine‐based therapies to treat a wide range of human diseases.

## Conflict of interest

The authors declare no conflict of interest.

## Author contributions

All authors listed have made a substantial, direct and intellectual contribution to the work, and approved it for publication.

## Data Availability

Data sharing is not applicable to this article as no new data were created or analysed in this study.
